# Analysis of the Crystallization Kinetics and Thermal Stability of the Amorphous Mg_72_Zn_24_Ca_4_ Alloy

**DOI:** 10.3390/ma14133583

**Published:** 2021-06-26

**Authors:** Bartosz Opitek, Janusz Lelito, Michał Szucki, Grzegorz Piwowarski, Łukasz Gondek, Łukasz Rogal

**Affiliations:** 1 Faculty of Foundry Engineering, AGH University of Science and Technology, 30 Mickiewicza Street, 30-059 Cracow, Poland; bartek3185@wp.pl (B.O.); piwgrz@agh.edu.pl (G.P.); 2 Foundry Institute, Technische Universität Bergakademie Freiberg, 4 Bernhard-von-Cotta-Str., 09599 Freiberg, Germany; Michal.Szucki@gi.tu-freiberg.de; 3 Faculty of Physics and Applied Computer Science, AGH University of Science and Technology, 30 Mickiewicza Street, 30-059 Cracow, Poland; lgondek@agh.edu.pl; 4 Institute of Metallurgy and Materials Science of Polish Academy of Sciences in Cracow, 25 Reymonta Street, 30-059 Cracow, Poland; l.rogal@imim.pl

**Keywords:** amorphous MgZnCa alloy, metallic glasses, crystallization kinetics, thermal stability

## Abstract

The aim of this study was to analyze the crystallization of the Mg_72_Zn_24_Ca_4_ metallic glass alloy. The crystallization process of metallic glass Mg_72_Zn_24_Ca_4_ was investigated by means of the differential scanning calorimetry. The glass-forming ability and crystallization are both strongly dependent on the heating rate. The crystallization kinetics, during the isothermal annealing, were modelled by the Johnson–Mehl–Avrami equation. Avrami exponents were from 2.7 to 3.51, which indicates diffusion-controlled grain growth. Local exponents of the Johnson–Mehl–Avrami equation were also calculated. In addition, the Mg phase—being the isothermal crystallization product—was found, and the diagram of the time–temperature phase transformation was developed. This diagram enables the reading of the start and end times of the crystallization process, occurring in amorphous ribbons of the Mg_72_Zn_24_Ca_4_ alloy on the isothermal annealing temperature. The research showed high stability of the amorphous structure of Mg_72_Zn_24_Ca_4_ alloy at human body temperature.

## 1. Introduction

There have been recent investigations of various materials used in orthopedics [1,2,3]. These materials are among the biomaterials group that do not have negative influences on the human body. The basic requirements for these materials are: biocompatibility, corrosion resistance and a lack of toxic components such as nickel, copper, vanadium, etc. [4,5,6]. The most common materials not subject to biodegradation, such as e.g., austenitic steels, titanium or cobalt alloys are applied in orthopedics. Out of all orthopedic materials, austenitic steels are characterized by the lowest mechanical and corrosive properties [7]. It has been reported that 316L corrodes in the body environment and releases iron, chromium and nickel [8]. Moreover, it contains 12.0–15.0% of nickel, which can be toxic to the human body if released. There is evidence that high levels of nickel ions in tissues are the cause of genotoxic and mutagenic activities or, when in contact with the skin, are the cause of the most widespread contact allergy and cancer [9,10]. The first report of an allergic reaction to an orthopedic implant described an eczematous rash over a stainless-steel fracture plate [11]. Numerous similar observations were documented later, with symptoms of discomfort, erythema, swelling and skin changes in the area of the implant [12,13]. In addition, some patients exhibited general discomfort, fatigue or weakness. The accumulation of nickel in the body through chronic exposure can lead to lung fibrosis, cardiovascular and kidney diseases [10]. The maximum rate of nickel release, due to corrosion in patients who have implants made of nickel alloys, is estimated as 20 mg kg^−1^ day^−1^ [14]. Against this backdrop of mounting health hazards caused by nickel toxicity, nickel-free nitrogen containing austenitic stainless steels with lower toxicity to the human body are thought to be developed as the next generation of metallic implant materials. Nitrogen not only replaces nickel for austenitic structure stability but also much improves steel properties. Cobalt alloys have better mechanical and plastic properties [15], while titanium alloys have a high biotolerance and corrosion resistance but not very good mechanical properties [16].

Investigations are currently focused on looking for modern materials that can be applied in medicine as biomaterials used for joining and repairing broken bones that are simultaneously biocompatible [17], biodegradable [18] and have the appropriate mechanical properties. Contact with these materials should not cause any side effects upon coming into contact with human organisms [19]. Biomaterials in the metallic glasses group, which are completely soluble in human organisms, were obtained in recent years [20]. They contain indispensable elements for life and health such as magnesium (Mg), zinc (Zn) and calcium (Ca) [21]. It was also discovered that metallic glasses from the Mg–Zn–Ca alloy group maintain good mechanical properties and corrosion resistance [22,23].

Gu and others [24] investigated mechanical properties as well as corrosion resistance of the Mg–Zn–Ca alloys. The performed tests suggested their high compression strength—more than 500 MPa—which is three times higher than pure magnesium, while corrosion investigations indicated a high corrosion resistance. It may then be assumed that the amorphous nature of metallic glasses can provide a material with higher corrosion resistance and better mechanical properties such as high strength and hardness than crystalline materials. However, wide usage of amorphous alloys is limited due to their low plasticity. The formation of a composite structure with glassy and crystalline phases can increase plasticity [25]. Obtaining an amorphous structure in objects with a larger cross-section may, however, be very difficult. It should be mentioned here that materials with a mixed (amorphous-crystalline) structure may still have good plastic properties and anticorrosion resistance [26]. This is what makes understanding the crystallization kinetics of Mg-based metallic glasses so important.

Currently, researchers are focused on investigating crystallizations of metallic glasses [27,28,29,30]. To date, there have not been any precise investigations of the crystallization kinetics of Mg–Zn–Ca metallic glasses. This knowledge is needed to understand the transformation process of metallic glasses with an amorphous structure, characterized by a disordered arrangement of atoms, into a precisely ordered crystal lattice resulting from the crystallization process. Due to the amorphous structure in metallic glasses being unstable, the crystallization process in these materials occurs intrinsically. The beginning and end times of the crystallization process of these glasses depend on the material chemical composition and temperature. Shortening the duration of and the acceleration of the crystallization process can be achieved via isothermal annealing at increased temperatures [31,32,33]. Due to this heat treatment, it is possible to obtain in situ composites in which the matrix constitutes the amorphous phase while reinforcing the crystalline phase. The crystallization process in metallic glasses and how it can be controlled is still not fully clear, however. In order to explain the behavior of materials at increased temperatures and to recognize their thermodynamic stability, investigations of the crystallization kinetics of metallic glasses are carried out.

Hu and others [34], when investigating the crystallization kinetics of the Ca–Mg–Zn metallic glass by isothermal annealing, calculated the crystallization rate and heat amounts emitted in this process as a function of the isothermal annealing temperature. An increase in the isothermal annealing temperature and its related heat emission led to a faster crystallization process. The duration of the crystallization process of Ca–Mg–Zn metallic glass shortens from approximately 25 min for the isothermal annealing temperature of 389 K to approximately 4 min at 405 K. 

The aim of investigations presented in this paper is the analysis of the crystallization kinetics of metallic glass of the Mg–Zn–Ca type. To achieve that, the isothermal annealing of the Mg_72_Zn_24_Ca_4_ metallic glass was performed via differential scanning calorimeter (DSC), variable-temperature diffraction (XRD) and scanning electron microscopy (SEM) examination. The reason why this alloy was selected is its chemical composition, which is close to the eutectic point. It is well known that an alloy with a eutectic or approximately eutectic composition, unlike a hypoeutectic alloy, can be more easily transformed into metallic glass. This is due to the fact that the process requires a slower cooling rate for an alloy with a eutectic or approximately eutectic composition than for a hypoeutectic alloy.

## 2. Materials and Experimental Methods

In order to obtain the Mg_72_Zn_24_Ca_4_ alloy, magnesium, zinc and calcium with the purity of 99.9% were used. Then, the melting was carried out in a resistance furnace under argon as an inert gas. After melting the liquid alloy was cast into a steel mold to obtain a cylindrical sample with a diameter of 20 mm and a height of 50 mm. After machining, the final diameter of the sample for further testing was 10 mm. The sample was then melted by spinning technique under argon to obtain a ribbon (about 150 µm thick) of the amorphous structure. The crystallization kinetics of the amorphous alloy was investigated by continuous heating and isothermal annealing in the TA DSC Q20 under a flow of high-purity argon. In the case of continuous heating, the heating rates were from 5 to 80 K/min. In the case of isothermal annealing, the sample was heated at a rate of 80 K/min to a desired temperature (between 498 K and 513 K), held at this temperature for a certain period of time until the crystallization was complete, and then rapidly cooled to room temperature. X-ray diffraction was employed to the amorphous nature of the ribbon prepared in this way. Additional investigations of the crystallization kinetics of the Mg_72_Zn_24_Ca_4_ amorphous alloy were performed using variable-temperature diffraction (XRD). These tests were performed at the isothermal annealing temperature of 495 K. The X-ray diffraction (XRD) patterns were collected using Panalytical Empyrean diffractometer equipped with a Cu K_α_ X-ray tube. The nonambient temperature studies were performed in an Anton Paar HTK 1200N chamber. The position of the sample was corrected against thermal displacement, and the temperature stabilization was better than 0.2 K. The analysis of the distribution of magnesium, zinc, and calcium in the structure of the amorphous ribbon was performed with the use of a scanning electron microscope (SEM). The SEM measurements were done using the JEOL 5900 LV microscope. For X-ray microanalysis, the NORAN System 6 energy dispersive spectrometer (EDS) was used.

## 3. Results and Discussion

**Figure 1:**
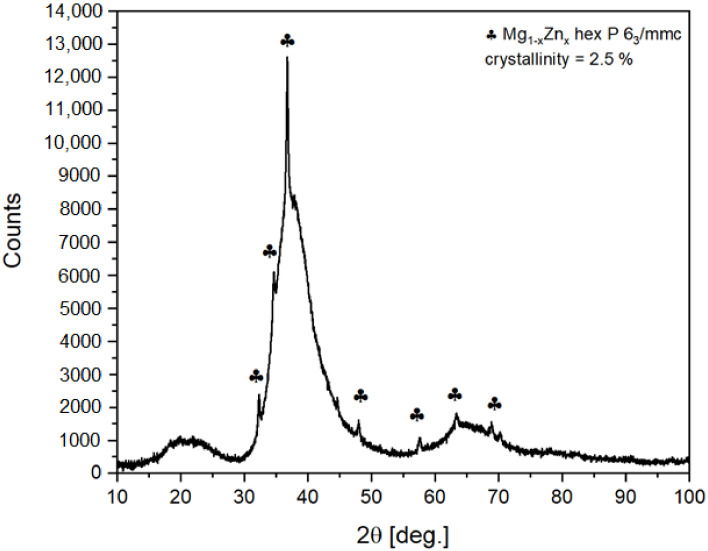
The X-ray diffraction pattern of Mg_72_Zn_24_Ca_4_ powdered ribbon.

Quantitative and qualitative phase analysis of the ribbon was performed. This means that the ribbon was pulverized. The XRD pattern of the powdered ribbon at 300 K is presented in Figure 1. Traces of crystal structure related to Mg_1−x_Zn_x_ with space group P6_3_/mmc were noticed. The crystallinity level was estimated to not be higher than 2.5%. The crystalline fraction of the sample was estimated by High Score Plus software. The procedure was as follows:

- measurement of the background originating from X-ray scattering on air and incident and diffracted beam optics (slits/collimators).
- Then, the sample was measured in the same way. The incident beam optics was set to enlighten just the sample surface and to exclude signal from holder.
- The background scan was then extracted from sample scan and procedure relaying on comparing the area under reflections to the total area was applied. The procedure could be applied as the amorphous and crystalline phases have the same composition according to EDS data. Therefore, correction for different scattering lengths was not required. The maximum of amorphous contribution is equivalent to interplanar distances of 2.9–1.9 Å. This correlates quite well with expected metallic radiuses of Mg, Zn and Ca, which are 1.6, 1.37 and 1.97 Å, respectively. Additional, smaller contribution at about 20° of 2θ angle (4.8–3.5 Å) seems to originate mainly from a distribution of Ca–Mg, Ca–Zn and Ca–Ca distances.

**Figure 2:**
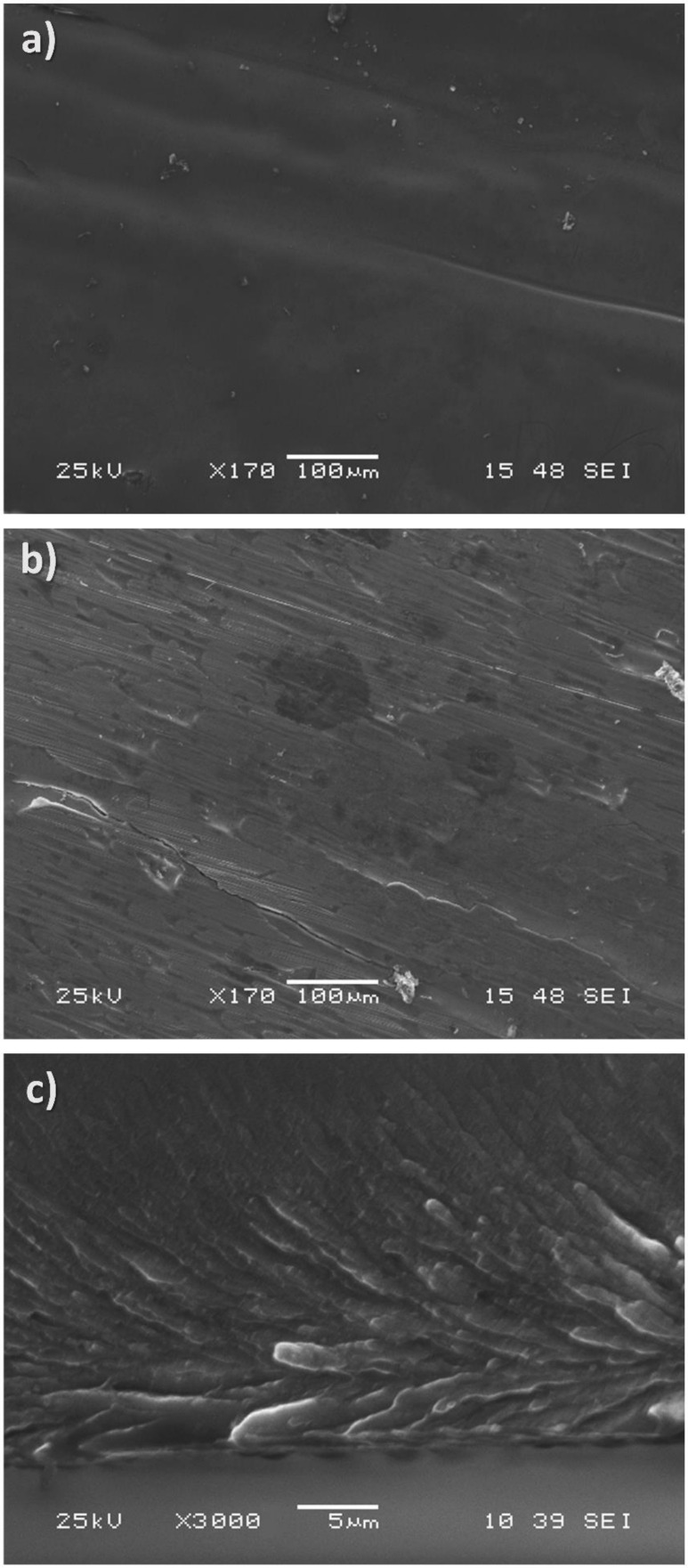
Electron microscopy studies of the Mg_72_Zn_24_Ca_4_ ribbon: glare side of the ribbon (**a**); matt (wheel) side of the ribbon (**b**); and fracture along the ribbon–matt side at the bottom (**c**).

In Figure 2, electron microscopy images of the ribbon are presented. As apparent, the wheel-side of the ribbon is quite different than the other side. Microstructure influenced by the copper wheel is clearly visible. Those images are typical for rapidly cooled ribbons. In Figure 2c, a cross-section of the ribbon along the spinning direction is presented. The ribbon was broken (not polished) under a protective Ar atmosphere to unveil all details of the microstructure. The bottom part (wheel side) exhibits an interesting microstructure reflecting centrifugal forces acting on the material during rapid solidification on the rotating copper wheel. Along with increasing distance from the bottom side, the microstructure changes into much smaller crystallites. The homogeneity is a much more apparent longer solidification time than for the bottom part.

**Figure 3:**
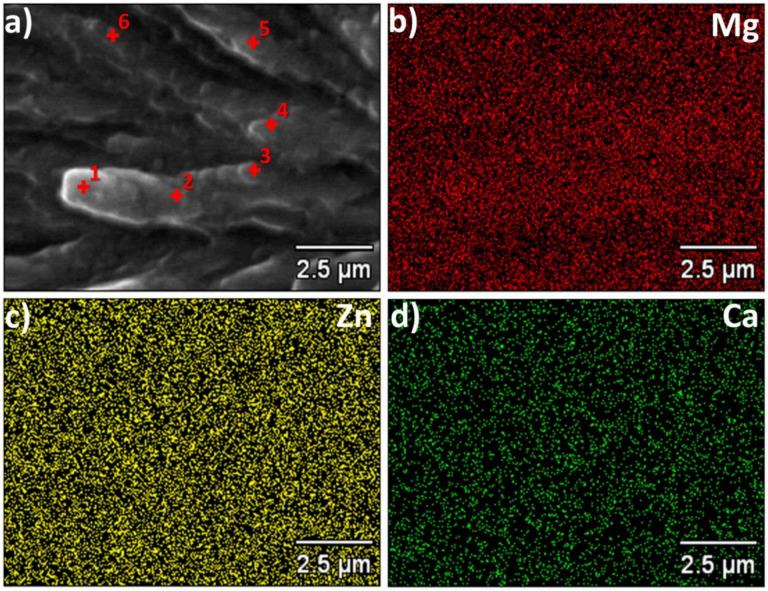
The X-ray microanalysis of the ribbon cross section with elemental distribution. For the marked points compositions are provided in Table 1. (**a**) SEM image with marked location of point analysis, (**b**) magnesium distribution, (**c**) zinc distribution and (**d**) calcium distribution.

Figure 3 shows the results of the X-ray microanalysis of the ribbon cross-section with magnesium, zinc and calcium distribution. According to Figure 3, the ribbon is quite homogeneous. Some small deviations were found at different points as gathered in Table 1.

Additionally, the EDS scans were made on broken ribbons as well as on both sides of it. The EDS map revealed that the composition in uniform across the entire sample.

**Figure 4:**
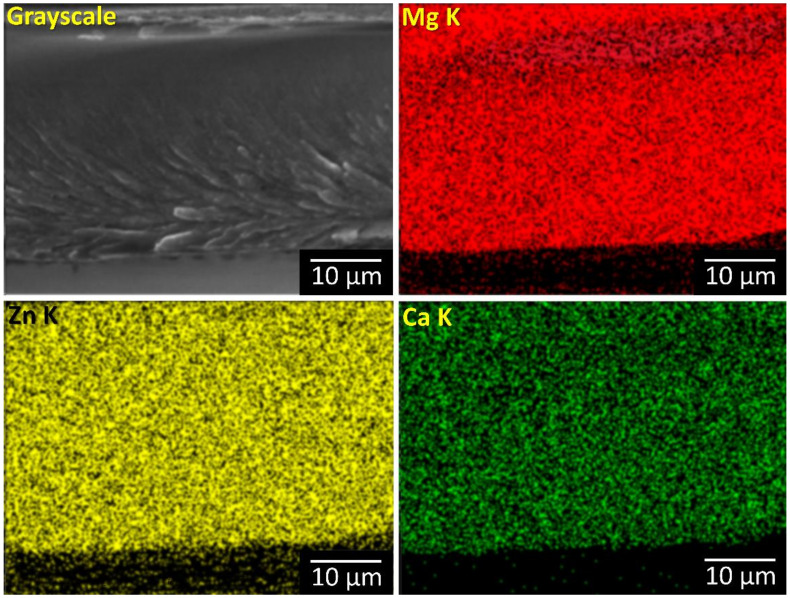
Cross section EDS map from large area of the broken ribbon.

Cross-section EDS map from a large area showed no inhomogeneities related to crystalline phases were noticed (Figure 4).

**Figure 5:**
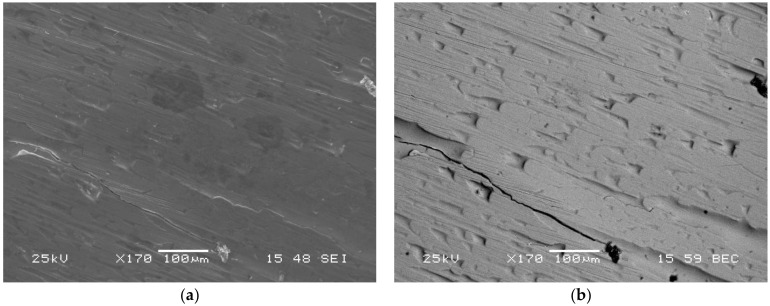
Images of the Mg_72_Zn_24_Ca_4_ ribbon both sides obtained using secondary electron detector (**a**) as well as backscattered electrons (element sensitive) (**b**).

For both sides of the ribbon the standard images using secondary electron detector (Figure 5a) as well as backscattered electrons (element sensitive) (Figure 5b) were made. One may compare both images of exactly the same place on the matt side of the ribbon (standard image using secondary electron on left, backscattered on right). Apparently, the ribbon shows extremely uniform distribution of backscattered electrons; hence, no compositional inhomogeneities were evidenced.

All this allowed us to conclude that the chemical compositions of crystalline and amorphous parts are the same.

**Figure 6:**
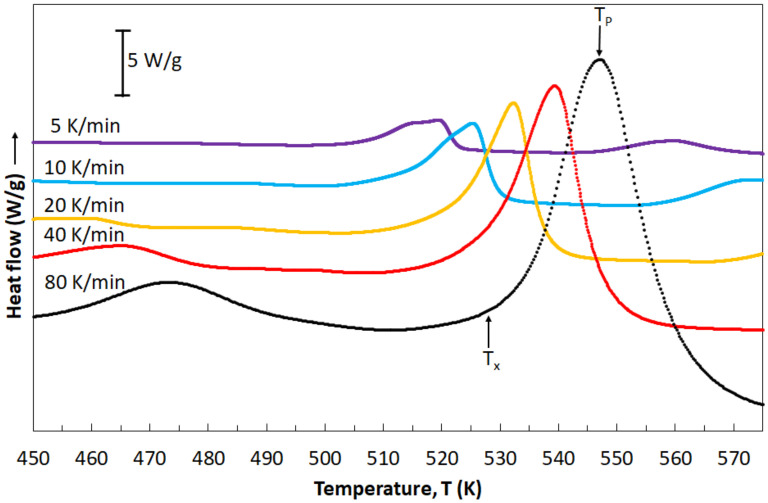
DSC curves of the amorphous Mg_72_Zn_24_Ca_4_ ribbons heated with the rates: 5, 10, 20, 40 and 80 K/min.

Ribbons of an amorphous structure (Mg_72_Zn_24_Ca_4_ alloy) were subjected to further investigations via differential scanning calorimetry (DSC). Investigations were based on continuous heating of ribbons with various rates: 5, 10, 20, 40 and 80 K/min. Figure 6 presents heat flow curves in dependence of heating rates. All DSC curves are characterized by the shape typical for metallic glasses. Peaks occurring in these curves allowed for the reading of various temperature values: temperature of the beginning of the crystallization process (*T*_x_) and temperature of the crystallization peak (*T*_p_). All these values are listed in Table 2. It is clearly seen that temperature values are increasing when the heating rates increase, indicating the glass-forming kinetics and a crystallization character. The influence of the heating rates on the crystallization process is caused by the thermal activation of the nucleation process, while the glass-forming kinetics is the result of relaxation processes within the glass transition region [35].

**Figure 7:**
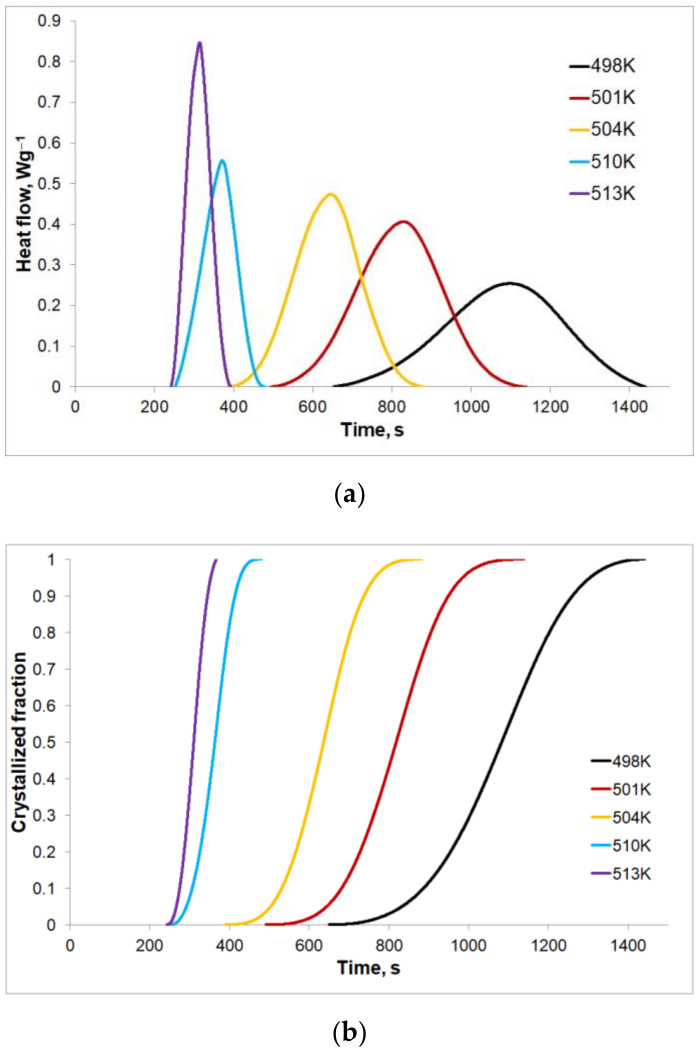
(**a**) Isothermal DSC curves and (**b**) the crystallized volumetric fraction as a time function for various isothermal annealing temperatures of Mg_72_Zn_24_Ca_4_.

The crystallization kinetics of amorphous ribbons of the Mg_72_Zn_24_Ca_4_ alloy at isothermal annealing for various temperature values: 498, 501, 504, 510, and 513 K were also tested and are presented in Figure 7. Figure 7a shows the heat flow rates for the given isothermal annealing temperatures. It can be noticed that all DSC curves indicate the individual endothermal peak after a certain time, called the incubation time. This time is shortened as the isothermal annealing temperature value increases. For the lowest value of the isothermal annealing temperature, the heat effect caused by the crystallization lasts the longest, at approximately 13 min with the peak height at its lowest. Along with an increase in the isothermal annealing temperature, the heat flows faster causing an increase in the peak height and shortening the crystallization process time to slightly above 2 min for the highest tested temperature of 513 K. On the bases of the isothermal annealing curves, changes of the crystallized volumetric fractions with an isothermal annealing temperature were determined. The results in the form of sigmoid curves are shown in Figure 7b. When comparing Figure 7a,b, it can be seen that the heat flow rate is proportional to changes of the crystallized volumetric fraction for the given value of the isothermal annealing temperature. Figure 7b also shows that the phase transformation duration, as well as the duration of the heat effect of the phase transformation, are different for different isothermal annealing temperatures. For the lowest isothermal annealing temperature (T = 498 K, Figure 7b), the phase transformation is the slowest and the longest (approx. 1441 s) compared to the phase transformations at higher isothermal annealing temperatures, where the end of the phase transformation occurs after approximately 1138 s for T = 501 K, 882 s for T = 504 K, 480 s for T = 510 K and 392 s for T = 513 K.

**Figure 8:**
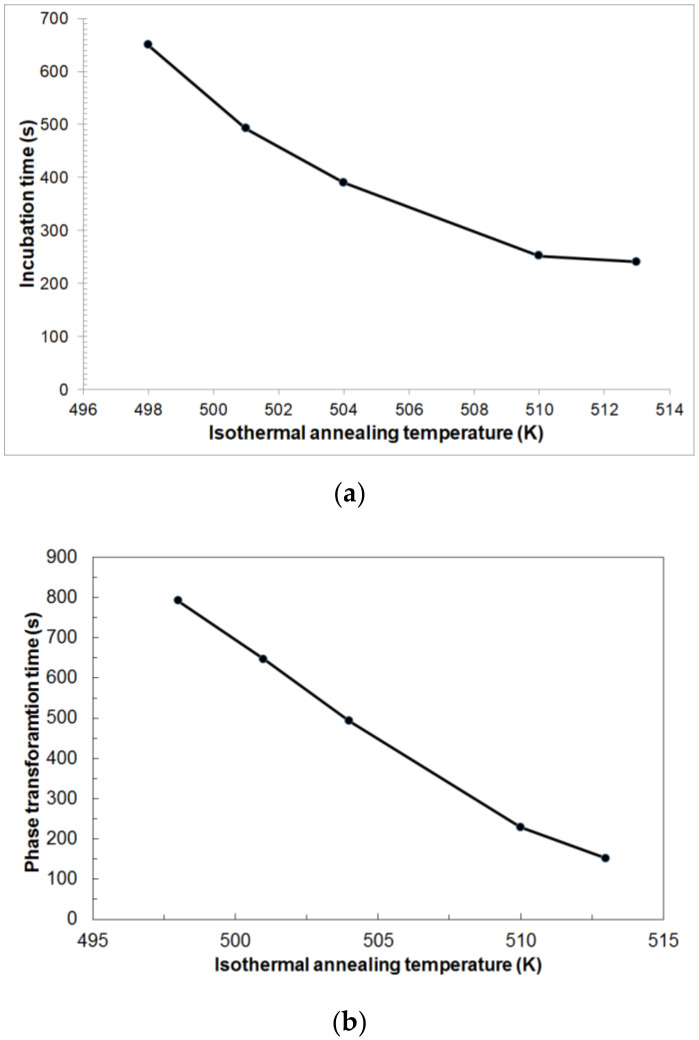
Incubation time (**a**) and duration of phase transformation (**b**) depending on the isothermal annealing temperature.

From the analysis of Figure 8a, certain incubation times are visible, depending on the isothermal annealing temperature, which are: 650 s for T = 498 K, 492 s for T = 501 K, 390 s for T = 504 K, 252 s for T = 510 K and 241 s for T=513 K. The incubation time decreases asymptotically with the increase in the isothermal annealing temperature. An increase in the isothermal annealing temperature shortens the duration of the heat effect and thus the actual duration of the phase transformation from 791 s for T = 498 K to 151 s for T = 513 K (Figure 8b). The phase-transformation time decreases in a nearly linear fashion with the increase in the isothermal annealing temperature. It can be stated that the increase in isothermal annealing temperature intensifies the kinetics of phase transformation.

**Figure 9:**
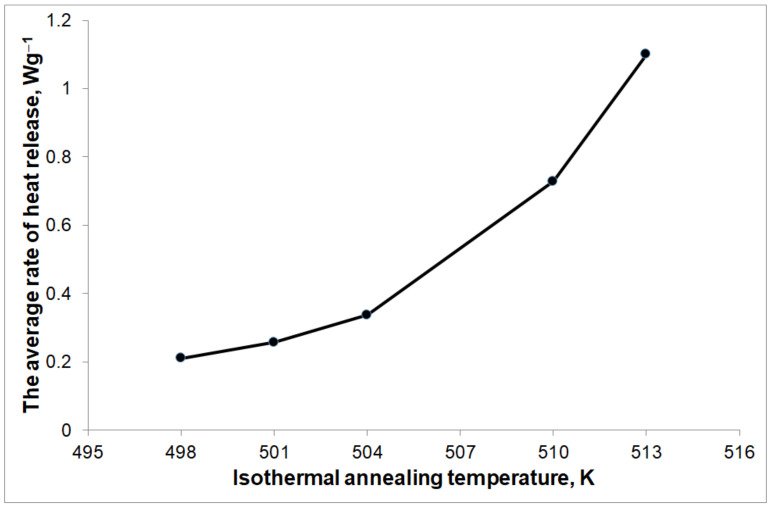
The average rate of heat effects release depending on the isothermal annealing temperature.

The amount of heat released during isothermal annealing was 166.2 Jg^−1^. The average rate of heat release (calculated as the quotient of the amount of heat released during the phase transformation to the duration of this transformation) during phase transformation depends on the isothermal annealing temperature (Figure 9) and is equivalent to the average nucleation rate and growth rate of the Mg_1−x_Zn_x_ phase. In the selected isothermal annealing temperature range of 498–513 K, the rate of nucleation and growth of this phase increases with the increase in the isothermal annealing temperature.

The crystallization kinetics of isothermal heating of the amorphous metallic Mg_72_Zn_24_Ca_4_ alloy was modelled by means of the Johnson–Mehl–Avrami (JMA) equation [36,37,38,39]:

(1) $$x=1-\exp\left[-k{\left(t-\tau \right)}^{n}\right],$$

where *x*—volumetric fraction of the crystallized phase, *t*—crystallization time, *τ*—incubation time, and *n*—Avrami exponent, which depends on the number of embryo formation and growth geometry. The value should be close to 4 when there is sporadic nucleation and a three-dimensional growth reaction takes place. In the case where *n* = 3, the transformation is only three-dimensional growth to predetermined nuclei. When the Avrami exponent is equal 2.5, the transformation occurs at a constant nucleation rate and the growth is controlled by diffusion, whereas when *n* = 1.5, the transformation is based only on the growth controlled by diffusion. The letter *k* in Equation (1) is the reaction rate constant, which depends on the nucleation kinetics and nucleus growth. Values of parameters *k* and *n* can be determined by means of the dependence described by Equation (1), which, after appropriate rearrangements, can obtain the following form:

(2) $$\ln\left(-\ln\left(1-x\left(t\right)\right)\right)=\ln k+n\ln\left(t-\tau \right).$$

**Figure 10:**
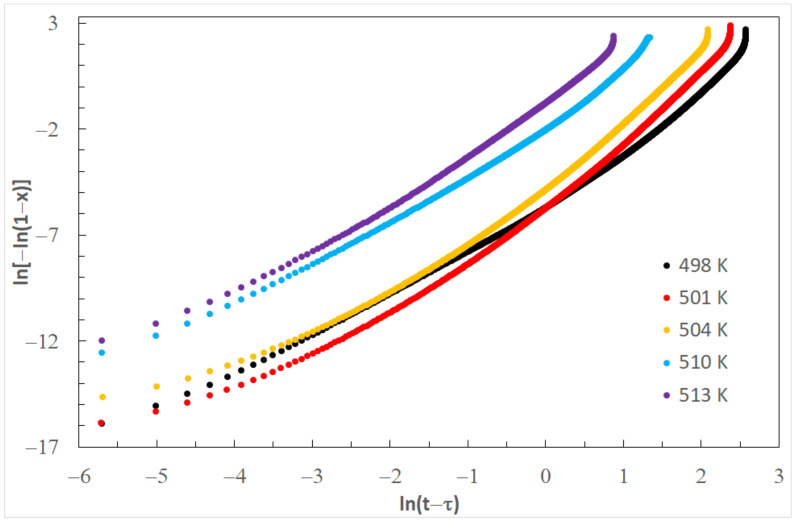
JMA curves for various isothermal annealing temperatures of the Mg_72_Zn_24_Ca_4_ alloy.

The JMA equation, rearranged into a form described by Equation (2), allows for the calculation of the *k* and *n* values. When drawing ln [−ln(1 − x)] versus ln(*t* − *τ*) for each temperature of isothermal annealing, the JMA diagram is obtained (Figure 10). Values of Avrami exponent *n* and the reaction rate constant *k,* determined on the basis of Figure 8, are listed in Table 3. Values of Avrami exponents are changing from 2.48 to 3.02, which means a diffusion-controlled increase in three directions [40].

**Figure 11:**
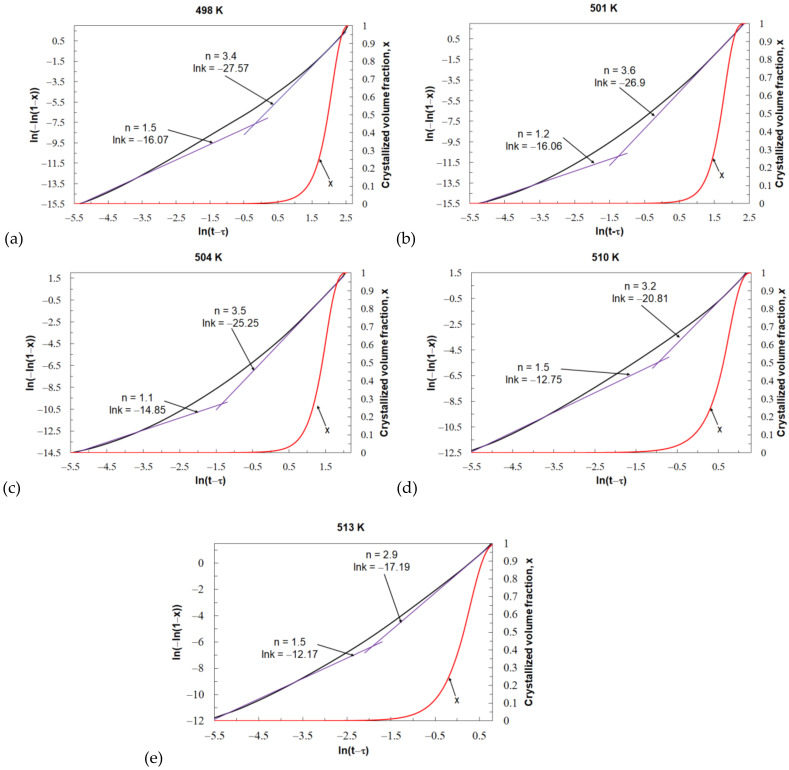
Avrami exponent and the crystallized volumetric fraction from the DSC analysis for Mg_72_Zn_24_Ca_4_ at different isothermal annealing temperatures, respectively, for: (**a**) 498, (**b**) 501, (**c**) 504, (**d**) 510 and (**e**) 513 K.

Figure 11 shows the JMA plots and the crystallized volume fraction for individual values of the isothermal annealing temperature of the tested Mg_72_Zn_24_Ca_4_ glassy alloy. For all values of the isothermal annealing temperature, the JMA plots are nonlinear. This means that the kinetic parameters *k* and *n* are time dependent. When investigating the crystallization of metallic glasses, the steady state of the process is taken into account, which usually corresponds to the volume fraction of the transformed phase in the range of 0.2–0.8. Within this range of the volume fraction of the transformed phase, the value of the Avrami exponent is relatively constant. In this case, the Avrami exponent *n* value changes from about 1 at the beginning of the crystallization process to about 3 and 3.5 at the end of the crystallization process. The value of the reaction rate constant *k* also changes during the crystallization process. The change in the value of the reaction rate constant *k* is the opposite of the change in the value of the Avrami exponent *n* during the crystallization process. For all isothermal annealing temperatures, the greatest value of *k* is at the beginning, and the lowest is at the end of the phase transformation. The specific values of the parameters *n* and *k* are shown in Figure 11. This phenomenon is related to the change in the nucleation process and the growth rate during the crystallization process. At the beginning of the crystallization process, where Avrami exponent *n* gave results over 1, the nucleation rate is very low, and the grain growth depends on diffusion processes. At the end of the crystallization process, where the calculated Avrami exponent *n* approaches and exceeds 3, there is a decreasing nucleation rate and controlled interface by growth. The values of *n* for beginning and end of the crystallization process look similar for each tested value of the isothermal temperature. This probably means that the mechanism of the crystallization process for the Mg_72_Zn_24_Ca_4_ metallic glass is similar at all values of the isothermal annealing temperature.

In order to investigate details of the crystallization process at a constant temperature, the local Avrami exponent *n*(*x*) was calculated by means of the equation [41]:

(3) $$n\left(x\right)=\frac{∆\ln\left[-\ln\left(1-x\right)\right]}{∆\ln\left(t-\tau \right)}.$$

**Figure 12:**
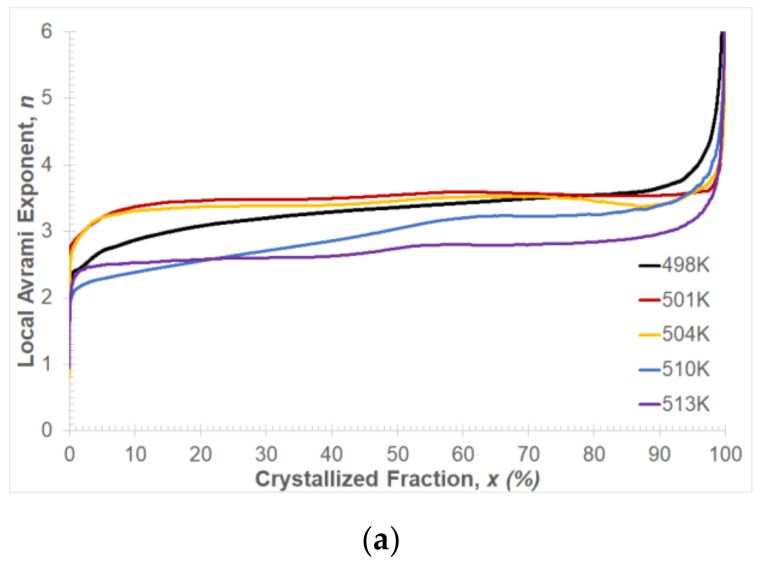

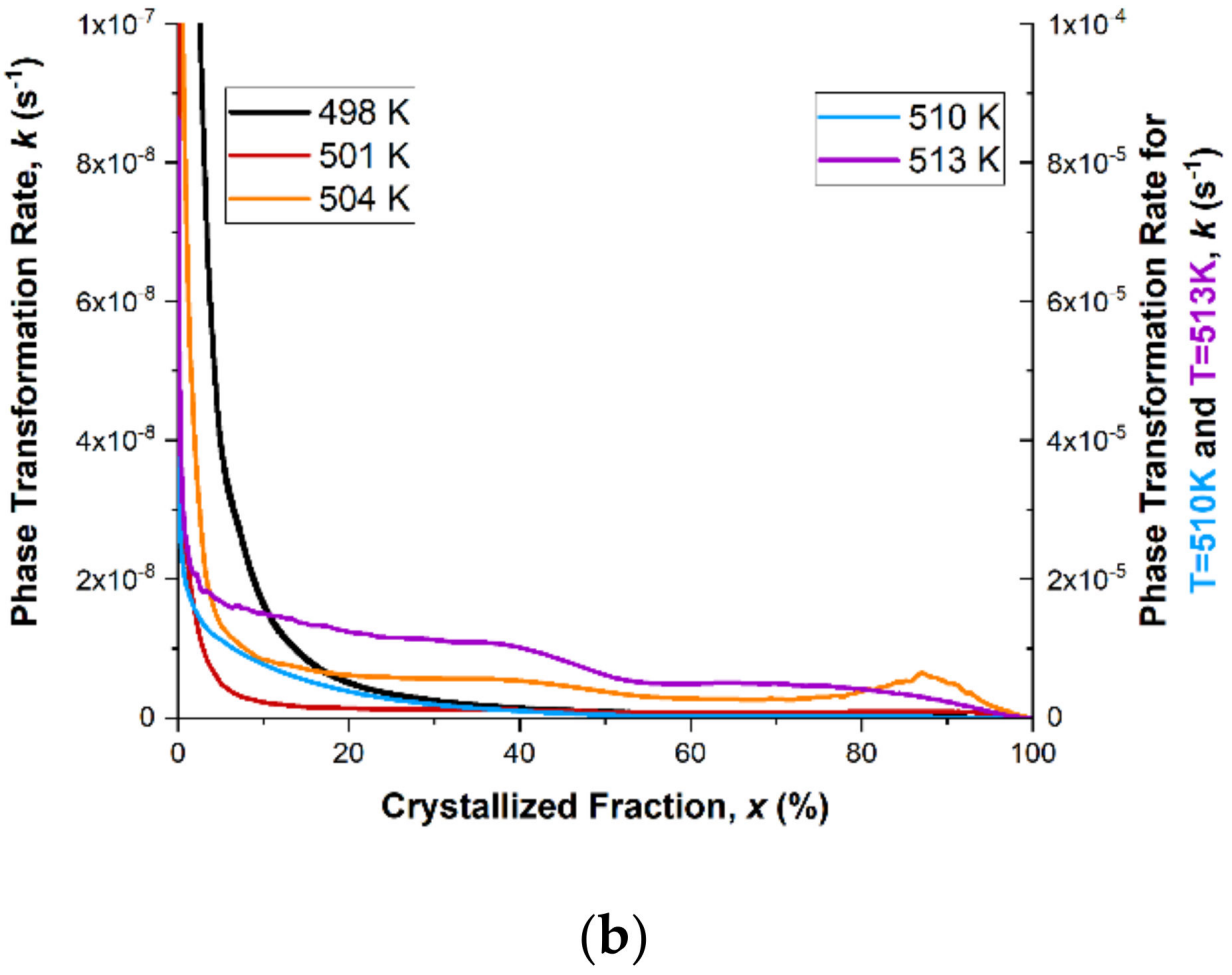
(**a**) Avrami exponent *n* and (**b**) reaction rate constant *k* in dependence on the crystallized phase amount, *x* (%).

The value of the local Avrami exponent *n* gives information about the nucleation process and the growth of the precipitates during the crystallization process. Figure 12a shows the results of the local Avrami exponent change as a function of the volume fraction of the crystallized phase. It can be observed that, for all temperature values of isothermal annealing within the crystallization range 10% < *x* < 90%, the local Avrami exponent values change from about 2.5 to 3.5. This change is related to the change in the course of the crystallization process depending on the values of the isothermal annealing temperature. For higher values of the isothermal annealing temperature, the transformation may occur at a constant nucleation rate and diffusion-controlled growth, while at lower values, the transformation process may be 3D growth and sporadic nucleation. At the end of the crystallization process, the Avrami exponent value increases rapidly. This behavior of the exponent *n* can cause the impingement effect [42], heterogeneous distribution of nuclei [43,44] and diffusion-controlled grain growth [45] in the Mg_72_Zn_24_Ca_4_ alloy.

Reaction constant *k* was calculated from Equation (2), and it describes the driving force of the reaction. The calculation results of the reaction rate constant *k* as a function of the crystallized fraction are presented in Figure 12b. A similar change in the value of the reaction rate constant *k* is visible for all values of isothermal annealing temperature. At the very beginning of the crystallization process, the reaction rate constant *k* has a very high value, which is related to the high value of the nucleation rate. Therefore, metallic glasses have heterogeneous nucleation sites, where nucleation is enhanced and the energy barrier lowered. The value of the reaction rate constant *k* drops very quickly, and at just 10% of the crystallized fraction, it reaches about 10^−8^ s^−1^. At this level, the value of the reaction rate constant *k* is kept to about 70% of the crystallized fraction. After 70% of the crystallized fraction, the rate of phase transformation increases and then drops to zero. This change in the reaction rate constant *k* applies to ribbons isothermally annealed at 498, 501 and 504 K. For ribbons isothermally annealed at 507 and 510 K, the change in the reaction rate constant *k* is similar to the lower isothermal annealing temperatures. At the very beginning of the crystallization process, a decrease in the value of the reaction rate constant *k* is also visible, while the kinetics of this decrease are slower, and the level of stabilization is achieved with the value of *k* equal to about 10^−5^ s^−1^. This means that at the very beginning of the phase transformation, the crystallization process is faster than for lower temperatures and is associated with a high nucleation rate. Additionally, the peak that appears at the end of the crystallization process is elongated and flattened. The change in the reaction rate constant *k*, and hence the rate of the phase transformation, which occurs at the end of the crystallization process, can be explained by the decreasing amount of the amorphous phase between the grains already in contact.

**Figure 13:**
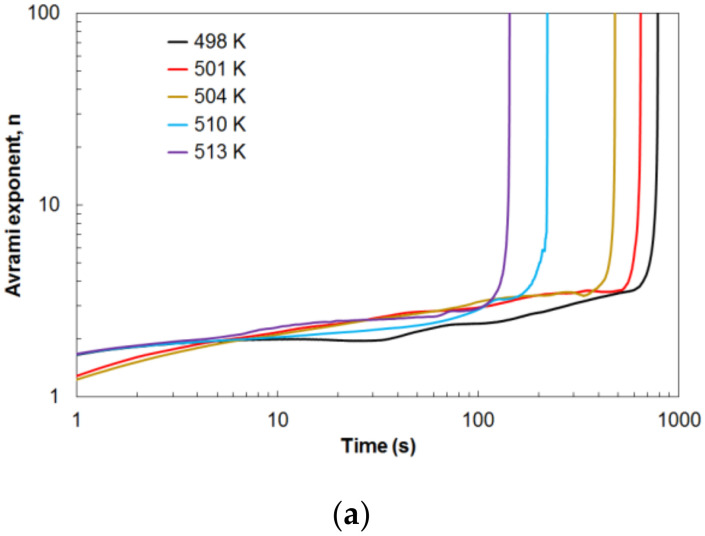

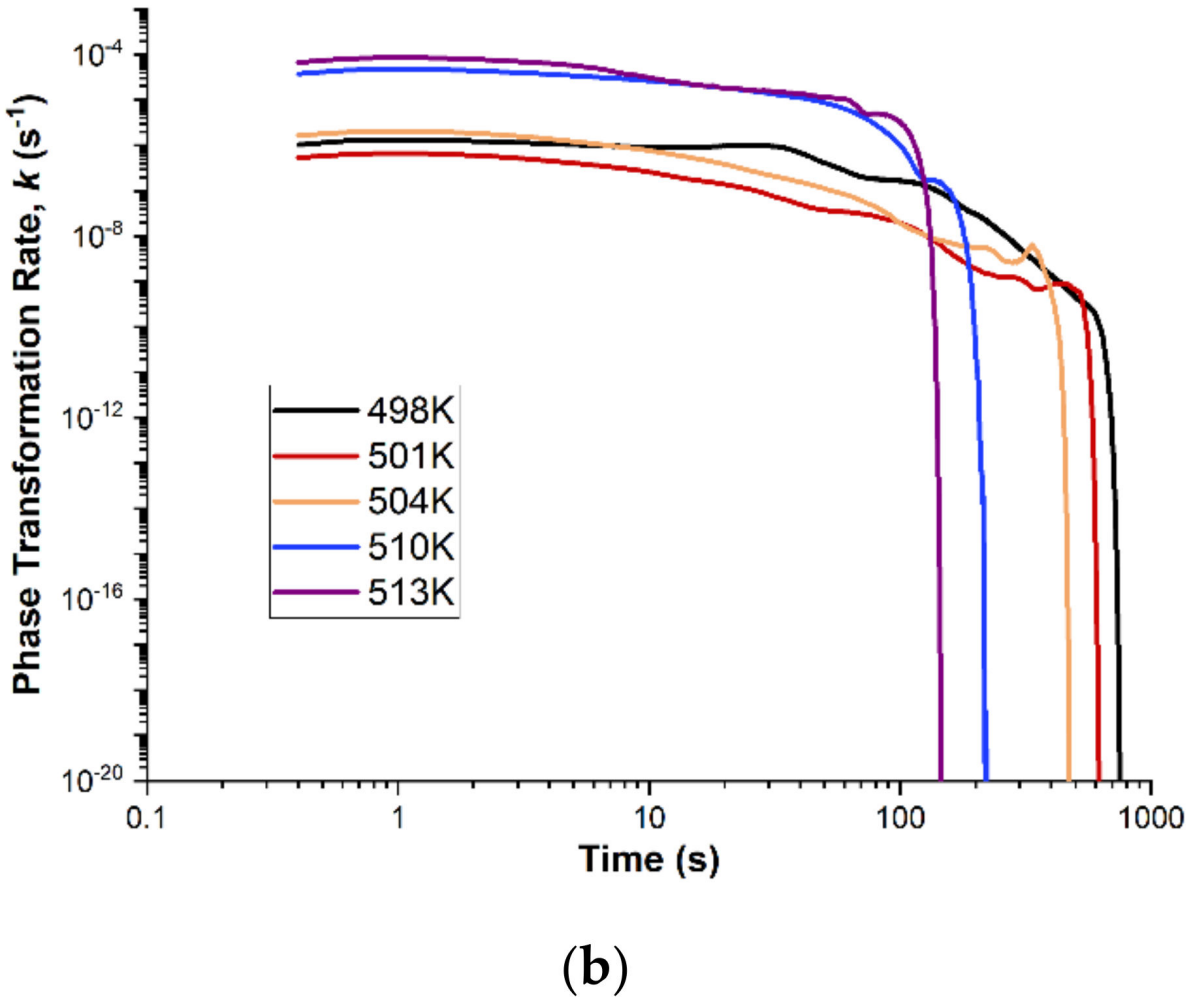
Variation of the (**a**) Avrami exponent *n*, and (**b**) reaction rate constant *k*, in time during the crystallization process in different annealing temperatures.

Earlier, it was shown how the Avrami exponent changes during crystallization. Figure 13a shows the change in values of the Avrami exponent *n* over time for different values of the isothermal annealing temperature. The Avrami exponent increases slowly in an almost linear fashion throughout the isothermal annealing. Only at the end of this process is a rapid increase in the value of the Avrami exponent *n* visible. This phenomenon is related to the end of the crystallization process. The crystallization time depends on the value of the isothermal annealing temperature. The higher the temperature value, the earlier the crystallization process ends, and the shorter the duration of the crystallization process. Therefore, a higher tested temperature means a faster crystallization process. Figure 13b shows the change in the value of the reaction rate constant *k* as a function of the transformation duration. The value of the reaction rate constant *k* is practically kept constant with a slight linear decrease during the crystallization process. At the end of the crystallization process, a sharp decrease in the value of the reaction rate constant to zero is visible. This phenomenon is related to the depletion of the amorphous phase at the end of the crystallization process. Moreover, the tests carried out for different isothermal annealing values showed different initial values of the reaction rate constant *k*. For each tested temperature, the course of the change in the value of the reaction rate constant *k* is similar. Higher isothermal annealing temperature also means an earlier sharp drop in the value of the reaction rate constant *k* to zero. This is due to the higher crystallization rate for higher temperatures.

**Figure 14:**
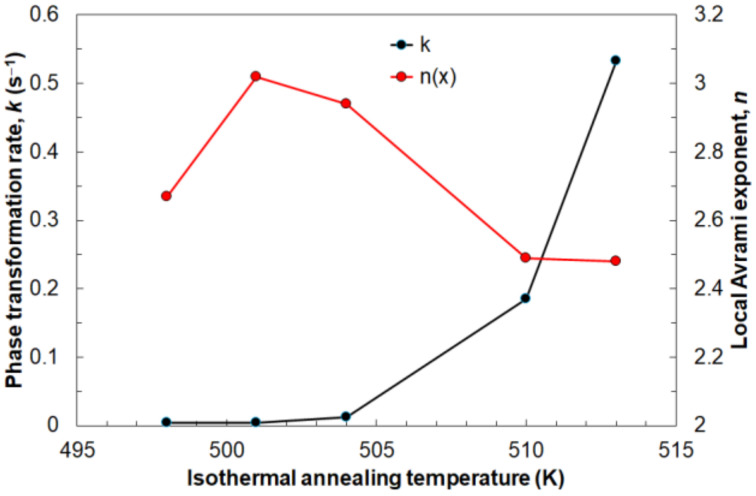
Relationship between reaction rate constant *k* and local Avrami exponent *n* for different isothermal annealing temperatures.

Figure 14 shows the influence of the annealing temperature on the average values of the Avrami exponent *n* and the reaction rate constant *k*. The Avrami exponent *n* increases at the beginning to reach a maximum value of about *n* = 3 at annealing temperature 501 K and then decreases to *n* = 2.5 at 510 K. The lowest tested temperature means a slow nucleation rate and depends mainly on diffusion. At 501 K, the highest nucleation rate was noted, after which it begins to decrease. This is the result of growth controlled by the interface. The reaction rate constant *k* constantly increases with the isothermal annealing temperature. This is the effect of the aforementioned faster crystallization process at higher values of temperature; therefore, the driving force of transformation is higher every subsequent tested temperature.

**Figure 15:**
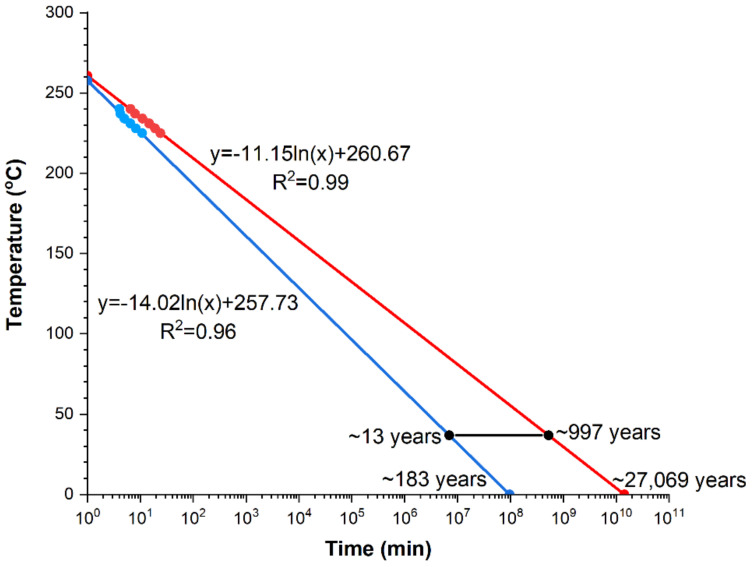
Diagram of time–temperature transformation for Mg_72_Zn_24_Ca_4_, together with marked human body temperature at 36.6 °C.

Investigations of the crystallization kinetics of the amorphous Mg_72_Zn_24_Ca_4_ also allowed for the determination of trend lines, suitable for predicting the times of the crystallization process at both the beginning and end for various temperatures of isothermal annealing (Figure 15). Both lines described by the equations: *y* = −14.02ln(*x*) + 257.73 and *y* = −11.15ln(*x*) + 260.67 for the crystallization beginning and end, respectively, were determined on the bases of the previously investigated-by means of DSC-points of the crystallization beginning and end. Determination coefficients are equal respectively: for the beginning of the crystallization process (R^2^ = 0.96) and the end (R^2^ = 0.99). According to expectations, the crystallization process is longer for lower temperature values of isothermal annealing. Since investigations were performed on the biodegradable amorphous alloy, the beginning and end of the crystallization process of the tested material at the human organism temperature, i.e., 36.6 °C, is marked by the black line in Figure 15. The expected beginning of crystallization under these conditions, occurs after approximately 13 years, while the end after nearly a thousand. Thus, the crystallization process at this temperature is very long.

**Figure 16:**
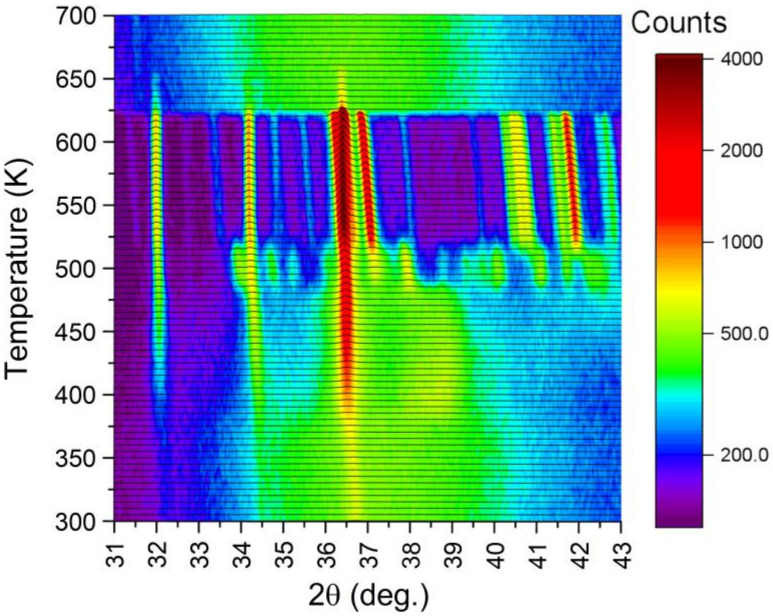
The X-ray diffraction patterns collected at nonambient temperatures.

**Figure 17:**
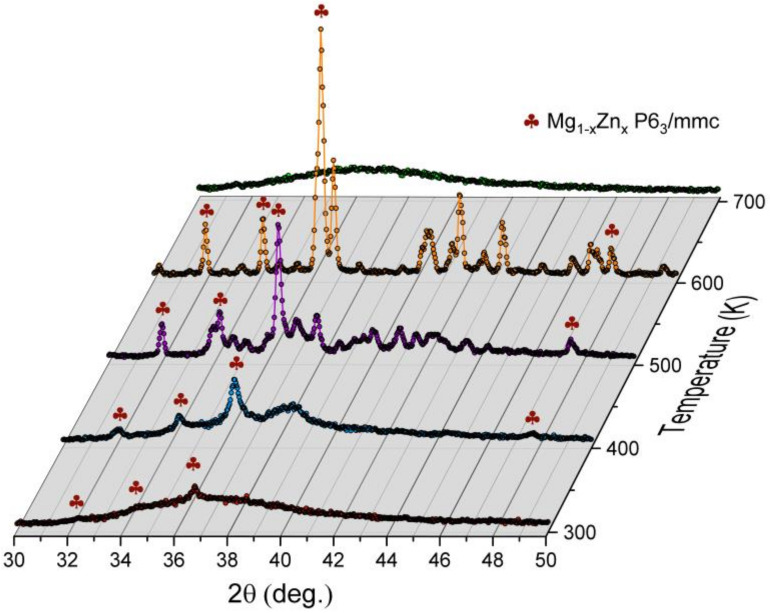
Examples of diffraction patterns collected at high temperatures.

Additionally, XRD studies were carried out to identify phase components that appear while heating the amorphous ribbon to the temperature of 700 K. The kinetics of crystallization of the amorphous ribbon were also tested using the same technique for the temperature of 495 K. In Figure 16, nonambient XRD patterns collected between 300–700 K are gathered. The temperature of 5 K was approached within 5 min, followed by 5 min for stabilization and 20 min for measurement. The mean heating ratio was quite slow, yielding only $\frac{1}{6}$ K/min. As apparent, the amorphous structure is stable up to 385 K. Above that, value reflections of the hexagonal Mg_1−x_Zn_x_ phase begin to develop. The association of those reflections is presented in Figure 17. At the same time, the amorphous contribution is diminishing. Interesting behavior can be noticed around 38.5° of 2θ angle. Namely, when the Mg_1−x_Zn_x_ hexagonal phase forms, there appears a broad reflection starting from 385 K. This contribution is visible up to 480 K, where a number of small intensity reflections appear. It is worth noting that at this temperature, the amorphous contribution is lost, which is reflected in the significant decrease in the background level. At 515 K, another phase transition is observed, where aforementioned small reflections turn into a few well-defined ones with high intensities. This behavior is also clearly seen in Figure 17, when one compares patterns collected at 500 and 600 K. The sample started to melt at 620 K, and above that temperature, only some traces of the Mg_1−x_Zn_x_ phase are visible. Those traces, which persist up to 655 K, are probably related to the Mg-rich Mg_1−x_Zn_x_ phase. In the melted state, the diffraction pattern resembles the one collected at 300 K, with some shift toward lower angles with regard to thermal expansion.

**Figure 18:**
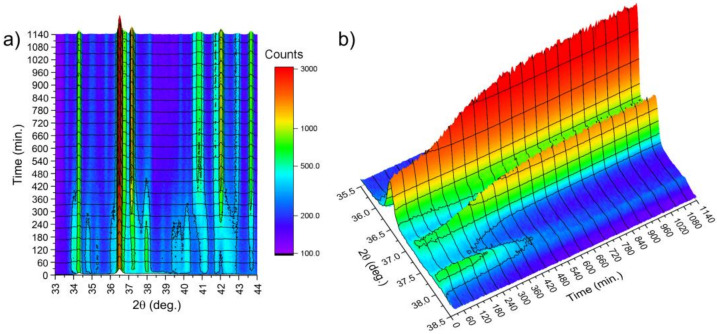
Evolution of XRD patterns with time at temperature of 495 K. (**a**) 2D view, (**b**) 3D view.

In order to examine the kinetics of the crystallization, the time-dependent evolution of XRD patterns was studied. The results are presented in Figure 18, where changes in diffraction patterns collected at 495 K were plotted. The target temperature was approached with the ratio 80 K/min, after which the acquisition of patterns began. The acquisition time was 55 s with 5 s for positioning of the diffractometer. Temporal resolution of 60 s was achieved. It was observed that reflections due to the Mg_1−x_Zn_x_ hexagonal phase appeared immediately, while reflections at the range of 37–38.5° of 2θ angle appeared after just 14 min (840 s). This was a clear indication that the crystallization process occurs. This time, called the incubation time, correlates with the value of the incubation time obtained with the differential scanning calorimeter shown in Figure 8a. After 600 min, no further changes were noticed, and diffraction patterns of the well-crystallized specimen were observed. The evolution of XRD patterns with time and thus the crystallization kinetics of the amorphous Mg_72_Zn_24_Ca_4_ alloy have a sigmoid curve shape and are similar to the kinetic phase-transformation curves shown in Figure 7b.

In summary, the amorphous structure is metastable. This means that it spontaneously changes into a stable crystal structure. The time of initiating the crystallization process and its duration depend mainly on the chemical composition of the alloy and the temperature of the medium. The structure of the alloy (amorphous, crystalline or mixed), apart from the chemical composition of the alloy, has a decisive influence on the corrosion resistance. The results of the corrosion resistance tests on the Mg_72_Zn_24_Ca_4_ alloy with an amorphous and crystalline structure are included in the literature [26]. These studies show that the amorphous structure, due to the lack of grain boundaries, is more resistant to corrosion in Ringer’s solution than the same alloy with a crystal structure only. Additionally, these samples, which lie midway between purely crystalline and purely amorphous in atomic structure, do not only exploit the coveted corrosion resistance of the amorphous structure but also enable processing of larger-sized samples sufficient enough to manufacture multitudes of biomedical devices.

## 4. Conclusions

The XRD investigations of ribbons indicated the amorphous structure of the Mg_72_Zn_24_Ca_4_ alloy with a small amount of the crystalline Mg_1−x_Zn_x_ phase. The kinetics of the crystallization process in amorphous ribbons were tested by means of DSC by heating and isothermal annealing at high temperatures. It was possible to read and compare the temperature at the beginning of crystallization (*T*_x_) and the temperature of the crystallization peak (*T*_p_) for various heating rates. The heating of ribbons of the amorphous Mg_72_Zn_24_Ca_4_ alloy at increased temperatures leads to increased rates of the heat emission and crystallization process course.

Holding the ribbons at the isothermal annealing temperature leads to intense heat generation. The average rate of heat release during nucleation and the growth of the magnesium phase increase with the increase in the isothermal annealing temperature (Figure 9). This phenomenon is probably caused by an increase in the rate of zinc diffusion in the amorphous magnesium matrix at higher isothermal annealing temperatures.

An increase in the isothermal annealing temperature causes a shortening of the phase-transformation times from 791 s for T = 498 K to 151 s for T = 513 K (Figure 8b). Isothermal annealing temperature also has a similar effect on incubation time, causing its shortening from 650 s for T = 498 K to 241 s for T = 513 K (Figure 8a).

The crystallization process course was modelled by means of the Johnson–Mehl–Avrami equation. The results of this modelling explicitly indicate that the crystallization process is controlled by a diffusional grain growth at increasing nucleation rates. The high value of reaction rate constant *k* in relation to the low value of Avrami exponent *n* can lead to rapid nucleation at the beginning of the process and, therefore, a larger transformed fraction than expected for purely uniform nucleation. This phenomenon can be explained by the fact that each metallic glass has a place for heterogeneous nucleation, in which nucleation is strengthened and the energy barrier is lowered. In addition, it should be mentioned that the amorphous alloy at the beginning of isothermal annealing already had a small amount of crystalline phase, which in addition could increase the concentration of the solidified volume through growth.

The obtained results also confirmed that the crystallization process of the amorphous Mg_72_Zn_24_Ca_4_ alloy at the human body temperature (36.6 °C) is very long. Its beginning occurs after 13 years, while the end only after a thousand years. Thus, on this basis, it can be assumed that the amorphous Mg_72_Zn_24_Ca_4_ is a stable material at the temperature of 36.6 °C. Usually, the crystalline structure is less resistant to corrosion than amorphous. This therefore means that not only the human body temperature but also the environment of bodily fluids influence degradation rates of this material.

## Figures and Tables

**Table 1:** Atomic composition of the Mg_72_Zn_24_Ca_4_ ribbon. Points 1–6 are marked in Figure 3, while area data were collected from an area of about 30 × 100 µm.

Number of Point	Mg (at.%)	Zn (at.%)	Ca (at.%)
1	74.1	22.7	3.2
2	70.5	25.9	3.6
3	73.0	23.2	3.5
4	73.5	23.5	3.0
5	68.9	27.0	4.1
6	67.6	28.6	3.8
Full area	70.2	25.9	3.7

**Table 2:** Characteristic temperature values (*T*_x_, *T*_p_) for the Mg_72_Zn_24_Ca_4_ alloy heated with the rates: 5, 10, 20, 40 and 80 K/min.

Heating Rate (K/min)	*T*_x_ (K)	*T*_p_ (K)
5	506	519.6
10	513	525.2
20	519	532.2
40	524	539.4
80	528	547.0

**Table 3:** Kinetic parameters (***n***, ***k***) for various temperatures of isothermal annealing.

Annealing Temperature (K)	Incubation Time, *τ* (min)	Avrami Exponent, *n*	Reaction Rate Constant, *k*
498	10.84	2.7	0.004
501	8.19	3.0	0.004
504	6.51	2.9	0.012
510	4.20	2.5	0.185
513	4.03	2.5	0.533

## Data Availability

The data presented in this study are available on request from the corresponding author.
